# Comparing frailty measures in their ability to predict adverse outcome among older residents of assisted living

**DOI:** 10.1186/1471-2318-12-56

**Published:** 2012-09-14

**Authors:** David B Hogan, Elizabeth A Freiheit, Laurel A Strain, Scott B Patten, Heidi N Schmaltz, Darryl Rolfson, Colleen J Maxwell

**Affiliations:** 1 Division of Geriatric Medicine, Faculty of Medicine, University of Calgary, HSC 3330 Hospital Drive NW, Calgary, AB, Canada; 2 Department of Community Health Sciences, University of Calgary, 3rd Floor TRW, 3280 Hospital Drive NW, Calgary, AB, Canada; 3 Department of Sociology, University of Alberta, 5-21 HM Tory Building, Edmonton, AB, Canada; 4 Division of Geriatric Medicine, Faculty of Medicine, University of Alberta, 1-108, 11350-83 Avenue, Edmonton, AB, Canada; 5 Schools of Pharmacy and Public Health & Health Systems, University of Waterloo, 200 University Avenue West, Waterloo, ON, Canada; 6 Institute for Clinical Evaluative Sciences, Toronto, ON, Canada

**Keywords:** Frailty, Predictive accuracy, Agreement, Assisted living

## Abstract

**Background:**

Few studies have directly compared the competing approaches to identifying frailty in more vulnerable older populations. We examined the ability of two versions of a frailty index (43 vs. 83 items), the Cardiovascular Health Study (CHS) frailty criteria, and the CHESS scale to accurately predict the occurrence of three outcomes among Assisted Living (AL) residents followed over one year.

**Methods:**

The three frailty measures and the CHESS scale were derived from assessment items completed among 1,066 AL residents (aged 65+) participating in the Alberta Continuing Care Epidemiological Studies (ACCES). Adjusted risks of one-year mortality, hospitalization and long-term care placement were estimated for those categorized as frail or pre-frail compared with non-frail (or at high/intermediate vs. low risk on CHESS). The area under the ROC curve (AUC) was calculated for select models to assess the predictive accuracy of the different frailty measures and CHESS scale in relation to the three outcomes examined.

**Results:**

Frail subjects defined by the three approaches and those at high risk for decline on CHESS showed a statistically significant increased risk for death and long-term care placement compared with those categorized as either not frail or at low risk for decline. The risk estimates for hospitalization associated with the frailty measures and CHESS were generally weaker with one of the frailty indices (43 items) showing no significant association. For death and long-term care placement, the addition of frailty (however derived) or CHESS significantly improved on the AUC obtained with a model including only age, sex and co-morbidity, though the magnitude of improvement was sometimes small. The different frailty/risk models did not differ significantly from each other in predicting mortality or hospitalization; however, one of the frailty indices (83 items) showed significantly better performance over the other measures in predicting long-term care placement.

**Conclusions:**

Using different approaches, varying degrees of frailty were detected within the AL population. The various approaches to defining frailty were generally more similar than dissimilar with regard to predictive accuracy with some exceptions. The clinical implications and opportunities of detecting frailty in more vulnerable older adults require further investigation.

## Background

Assisted living (AL) is typically defined as a setting that provides health and personal services within a secure residential environment for older adults not requiring the continuous monitoring and more intensive professional care of a long-term care facility [1,2]. It is a growing and in many ways attractive housing option for older persons often described as frail. AL residents are prone to a number of adverse outcomes in the short term. Over a year approximately a sixth will die with a similar proportion moving to a higher level of care [3,4]. Acute care hospitalizations also occur frequently among this group of older adults [4,5].

There is consensus that the core feature of frailty is a heightened vulnerability to stressors (for a given chronological age and sex), which leads to an increased risk for multiple adverse health-related outcomes [6]. As a group, AL residents will have relatively higher levels of vulnerability than similarly aged community-dwelling individuals, but within the AL population there will be varying degrees of frailty. Gradients of frailty are found in long-term care populations with higher levels associated with an increased risk of mortality, cognitive decline, and new onset disability [7]. Identifying AL residents at a higher risk for adverse health outcomes would offer opportunities to both maintain remaining independence and enhance quality of life if this could be linked to effective interventions.

Few studies have directly compared the competing approaches to identifying frailty in more vulnerable older populations. Two frequently used measures are the *frailty index*, which is based on a count of accumulated deficits divided by the number of potential deficits [8], and the *Cardiovascular Health Study (CHS) frailty criteria*. With the CHS approach, the determination of frailty requires the presence of three or more characteristics felt to capture what has been termed the phenotype of frailty [9].

Data arising from the implementation of the interRAI family of assessment instruments have increasingly been used for aging-related research, including recent work on frailty [10-13]. For example, Armstrong and colleagues developed a 50-item frailty index based on items in the home care instrument [10]. Embedded within each interRAI instrument are various scales. One of them, the Changes in Health, End-stage disease and Signs and Symptoms (CHESS) scale, is a measure of health stability designed to identify individuals at high risk for clinically significant decline [11]. In nursing home populations, higher CHESS scores are predictive of mortality and hospitalization [11,12]. Some have suggested that the CHESS scale is a frailty measure [11,13].

The primary objective of this study was to compare the ability of two versions of a frailty index derived from interRAI assessment items and the CHS frailty criteria to accurately predict the occurrence of three outcomes relevant to an AL population (mortality, hospitalization, and transfer to a long-term care facility) over a year. This builds on work previously done by our group in trying to operationalize the assessment of frailty among older residents of AL facilities [14]. A secondary objective was to compare these frailty measures to the CHESS scale in their ability to predict these three outcomes.

## Methods

### Study design

This was a sub-study of the Alberta Continuing Care Epidemiological Studies (ACCES), which investigated the health status of residents and quality of care issues within AL and nursing home facilities across the Province of Alberta, Canada. The AL cohort of ACCES consisted of residents aged 65 years and older residing in designated (i.e., publicly-funded) assisted living and supportive housing facilities (DAL) in two urban and three rural health regions within Alberta. The details of this study have been reported elsewhere [3,14]. A total of 1,089 AL participants were enrolled and assessed. This sub-study includes 1,066 residents who provided consent for linkage with Provincial administrative data and had a known outcome status during the one-year follow-up.

Ethics approval was obtained from the University of Calgary Conjoint Health Research Ethics Board, the University of Alberta Health Research Ethics Board and the University of Lethbridge Human Subject Research Committee. Administrative approvals from the health regions and/or facilities were also obtained. Informed consent was received from all participants or their legally appointed proxy decision-maker.

Trained research nurses administered the Resident Assessment Instrument for Assisted Living (interRAI-AL) and additional performance-based frailty measures at baseline (2006–2007) and at 1-year (when possible). The interRAI-AL tool is a comprehensive, standardized assessment of the sociodemographic characteristics, physical and cognitive status, health conditions, behavioural problems, and use of medications and services of residents [15].

### Frailty measures and CHESS score

Three frailty measures were examined. Their specific composition is described in Appendix A. The first was a frailty index based on the report of Armstrong et al. [10] that we designated as the *Armstrong Index* (see Appendix A). We employed a modified version with 43 of the original 50 items, as not all items were available with the interRAI-AL tool. An alternative frailty index consisting of 83-items derived from the interRAI-AL was created using the procedure described by Searle et al. [16], which we designated as the *Full Frailty Index* (see Appendix A). At least 30–40 items, fulfilling recommended criteria, are suggested for inclusion in a frailty index. In general, the more items included, the more precise the estimates of frailty obtained [16]. Our intent with the Full Frailty Index was to make maximal use of the interRAI-AL tool to develop a precise, practical index that was also substantially different from the Armstrong Index (i.e., 44 of the 83 items were not contained in the Armstrong Index). Both indices are calculated as the proportion of accumulated to potential deficits present in a given individual. For most items included in both indices, there was less than 5 percent missing data (with ≥ 80% of items having no missing values) [17]. For three variables (bathing, walking, self-reported health), there were higher percentages with missing values (6.9%, 11.6%, 11.6%, respectively) because either the activity did not occur during the three-day assessment time frame (bathing, walking) or the resident could/would not respond (self-reported health). These variables were retained in the respective indices (bathing and self-reported health in both indices; walking in the Full Frailty Index as well) as each individual variable makes a small contribution to the indices and prior work indicated that doing this would not significantly affect our results [16]. Any variable where there was a missing value was removed from both the numerator and denominator of the ratio used to calculate the frailty index for an individual participant (i.e., if one of our research subjects had one variable with a missing value, when calculating their Full Frailty Index the denominator was 82). In accord with other studies [16,18], a resident with a score of less than 0.2 was considered “robust” or “not frail” for both indices while scores between 0.2-0.3 (inclusive) indicated a pre-frail state and a score greater than 0.3 would lead to the resident being categorized as frail.

The determination of frailty using the CHS approach is based on the presence of five criteria: slow gait, muscle weakness, low physical activity, unintentional weight loss, and exhaustion. If none are present, the person is categorized as not frail [9]. The presence of three or more denotes the presence of frailty while one or two signifies pre-frail status. Two of these criteria are performance-based. Please see Appendix A. Nearly 40% of AL participants could not complete the CHS frailty assessment as originally intended. By using responses to observed items from the interRAI-AL assessment instrument we were able to reduce the proportion with missing data to 15% [14]. For all analyses using CHS criteria our sample size was reduced to 927.

The derivation of the CHESS scale followed standard interRAI procedures using items from the interRAI-AL (see Appendix B) [11]. Two symptoms (i.e., dehydration, decline in food/fluid intake) of the six included in the original CHESS scale were unavailable on the interRAI-AL form and not used in calculating the symptom component of the total score. Based on the distribution of the scores in our study population and our desire to keep the number of categories comparable to the frailty measures, we collapsed the typical five point scale and categorized the scores as follows: 0 indicating a low risk for serious decline, 1 as intermediate, and 2 or more denoting a high risk.

### Outcome measures and baseline characteristics

Primary outcomes were mortality, one or more hospitalizations and transfer to a long-term care facility over the 12 months following the subject’s baseline assessment. For mortality and long-term care admission, information on event occurrence was determined from concurrent reviews of facility discharge records and family interviews (at the time of death or discharge) as well as data collected at the time of resident and family follow-up assessments. For hospitalization, residents’ data were linked with the Alberta Inpatient Discharge Abstract Database for 2002–03 to 2008–09. This administrative database captures essentially 100% of all hospital admissions in the Province of Alberta.

A baseline co-morbidity score was calculated based on the Charlson co-morbidity index [19] and a validated coding algorithm [20] using relevant diagnostic codes (any occurrence during 3 years prior to baseline) from the Alberta Inpatient Discharge Abstract database. Other baseline characteristics examined included an additional co-morbidity measure (based on the sum of recorded diagnoses on the interRAI-AL) and three validated scales derived from items on the tool: the Cognitive Performance Scale (CPS) [21], Depression Rating Scale (DRS) [22], and Activities of Daily Living (ADL) Self-Performance Hierarchy Scale [23].

### Analysis

A weighted kappa statistic (with 95% confidence interval) was used to measure the agreement between the Armstrong Index, the Full Frailty Index, the CHS criteria, and the CHESS scale, using the 3-level risk categorization [24,25]. Generalized linear models with a binomial distribution and log link were used to estimate risk ratios in analyses. Multivariable models adjusting for age (in years), sex, and co-morbidity were examined to assess the prognostic significance of each approach to defining frailty and the CHESS scale. The risks of one-year mortality, hospitalization (one or more), and transfer to long-term care for those categorized as frail or pre-frail by the Armstrong Index, the Full Frailty Index, and CHS criteria (compared with non-frail residents) were assessed in multivariable models. For the CHESS scale, high and intermediate risk levels were compared to the low risk group. The models considered potential confounding by age, sex and co-morbidity (using the Charlson co-morbidity index score). Predictive accuracy was assessed by comparing the area under the receiver operating characteristic curve (AUC) of a baseline model with only age and sex with that of models with age/sex/co-morbidity and age/sex/co-morbidity/frailty (or CHESS), respectively. To facilitate model comparisons, 95% confidence intervals for differences in AUC estimates (with associated p-values) were calculated [26]. Combined outcomes, death/hospitalization and death/move to long-term care, were also explored to investigate the possibility of competing risks. As there were no appreciable differences in our final conclusions, the results were not included.

We previously examined the effects of clustering (i.e., violation of the independence assumption due to nesting by AL-institution) on the outcomes and determined that the level of clustering was minimal [14]. We have therefore presented the results of our main analysis without adjustment for clustering to permit simpler statistical approaches without the loss of information. SAS Version 9.2 (SAS Institute, Inc., Cary, NC) was used for analyses.

## Results

The enrolled cohort was predominantly female (818/1066 or 76.7%) with an average age of 84.9 (standard deviation 7.3) years (see Table 1). Multiple morbidities were common. Most subjects (n = 632, 59.3%) had at least mild cognitive impairment, nearly a fifth (n = 203, 19%) had significant depressive symptoms, and over a third (n = 426, 40%) had limited or greater impairment in the performance of activities of daily living.

**Table 1:** Baseline characteristics for ACCES – AL Cohort

**Characteristicn (%), unless otherwise noted**	**Main sample (n = 1,066)**	**CHS measurement (n = 927)**^**1**^
Age, mean ± SD	84.9 ± 7.3	84.9 ± 7.3
Female	818 (76.7)	700 (75.5)
Charlson co-morbidity index, mean ± SD	1.8 ± 2.0	1.8 ± 2.0
inter*RAI-AL* co-morbidity count, mean ± SD	4.65 ± 2.0	4.65 ± 1.95
Cognitive Performance Scale (CPS)		
Intact/borderline intact (score 0–1)	434 (40.7)	399 (43.0)
Mild impairment (score 2)	336 (31.5)	305 (32.9)
Moderate impairment (score 3–4)	183 (17.2)	151 (16.3)
Severe/very severe impairment (score 5–6)	113 (10.6)	72 (7.8)
Significant depressive symptoms (DRS 3+)	203 (19.0)	158 (17.0)
Activities of Daily Living (ADL) Scale		
Independent (score 0)	454 (42.6)	426 (46.0)
Supervision required (score 1)	186 (17.4)	160 (17.3)
Limited impairment (score 2)	126 (11.8)	106 (11.4)
Extensive assistance required (score 3–4)	243 (22.8)	197 (21.2)
Dependent (score 5–6)	57 (5.4)	38 (4.1)
**Armstrong Index**		
Not frail, score <; 0.2	99 (9.3)	
Pre-frail, score ≥ 0.2 and ≤ 0.3	410 (38.5)	
Frail, score > 0.3	557 (52.3)	
**Full Frailty Index**		
Not frail, score <;0.2	344 (32.3)	
Pre-frail, score ≥ 0.2 and ≤ 0.3	416 (39.0)	
Frail, score > 0.3	306 (28.7)	
**CHS Frailty Criteria**		
Not frail, score = 0		32 (3.5)
Pre-frail, score = 1,2		451 (48.7)
Frail, score = 3+		444 (47.9)
**CHESS Scale**		
Low risk of decline, score = 0	496 (46.5)	
Intermediate risk, score = 1	312 (29.3)	
High risk, score = 2+	258 (24.2)	

*ACCES* Alberta Continuing Care Epidemiological Studies, *AL* assisted living, *SD* standard deviation, *CHS* Cardiovascular Health Study.

^1^Excluded observations due to missing frailty criteria, not linking to hospital data, loss to follow-up.

On the Armstrong Index, 9.3% (n = 99) were categorized as not frail, 38.5% (n = 410) pre-frail, and 52.3% (n = 557) as frail. The Full Frailty Index categorized 32.3% as not frail (n = 344), 39% pre-frail (n = 416), and 28.7% (n = 306) as frail. Using CHS criteria, 3.5% (n = 32) were categorized as not frail, 48.7% (n = 451) pre-frail and 47.9% (n = 444) as frail. On the CHESS scale, nearly half (46.5%, n = 496) were identified as being at a low risk for a serious decline, with 29.3% (n = 312) and 24.2% (n = 258) categorized as intermediate and high risk respectively.

A weighted kappa statistic showed the highest level of agreement between the Armstrong Index and the Full Frailty Index (kappa = 0.41, 95% CI 0.38-0.45) with just over half (51.7%) having identical categorizations as non-frail, pre-frail and frail on both criteria (see Table 2). The poorest agreement was between the CHS frailty measure and the CHESS scale (kappa = 0.11, 95% CI 0.08-0.14) with only 30.4% assigned to equivalent categories (non-frail/low risk, pre-frail/intermediate risk, and frail/high risk).

**Table 2:** Comparison and assessment of agreement among the frailty measures, based on 3-level risk categorization

**Comparison**^**1**^	**Percent of residents with concordance in categorization**	**Weighted kappa**	**95% CI**
Armstrong Index – Full Frailty Index	51.7	0.41	0.38-0.45
Armstrong Index – CHS Frailty Criteria	57.5	0.29	0.24-0.34
Armstrong Index – CHESS Scale	34.4	0.15	0.12-0.19
Full Frailty Index – CHS Frailty Criteria	38.2	0.17	0.13-0.20
Full Frailty Index – CHESS Scale	51.7	0.36	0.31-0.40
CHS Frailty Criteria – CHESS Scale	30.4	0.11	0.08-0.14

^1^All comparisons based on n = 1066 residents except for comparisons involving the CHS Frailty Criteria where n = 927 residents.

Over a year 15.9% (n = 170) of residents died, 39.8% (n = 424) were hospitalized at least once, and 19.1% (n = 204) moved to long-term care (see Table 3). Frail subjects defined by the three approaches as well as those at a high risk for decline on the CHESS scale showed a statistically significant increase in their risk of dying compared to those categorized as either not frail or at low risk for decline. The risk ratios in models adjusted for age, sex, and co-morbidity ranged from 1.74 (95% CI 1.07-2.81) with CHS frailty criteria to 2.35 (95% CI 1.56-3.54) for the Full Frailty Index. Only the Full Frailty Index was associated with a statistically significant increase in the risk of death for pre-frail subjects in our adjusted models (RR 2.00, 95% CI 1.33-3.00).

**Table 3:** One-year outcomes* associated with frailty measures, Risk Ratios (95% Confidence Intervals)

**Frailty model**			**Death**	**Hospitalization**	**Move to Long-Term care**
			**(170 events)**^**1**^	**(424 events)**^**1**^	**(204 events)**^**1**^
**Armstrong Index**	Unadjusted	Prefrail	1.23 (0.63-2.44)	0.93 (0.69-1.24)	**2.25 (1.00-5.08)**
**N = 1,066**		Frail	**2.27 (1.09-4.32)**	1.23 (0.94-1.63)	**4.21 (1.91-9.25)**
	Adjusted^2^	Prefrail	1.17 (0.59-2.29)	0.91 (0.68-1.22)	2.21 (0.98-4.99)
		Frail	**1.94 (1.02-3.70)**	1.16 (0.87-1.53)	**4.14 (1.87-9.14)**
**Full Frailty Index**	Unadjusted	Prefrail	**2.22 (1.47-3.34)**	**1.44 (1.19-1.74)**	**1.85 (1.26-2.72)**
**N = 1,066**		Frail	**2.69 (1.78-4.07)**	**1.33 (1.09-1.63)**	**3.30 (2.30-4.75)**
	Adjusted^2^	Prefrail	**2.00 (1.33-3.00)**	**1.37 (1.13-1.66)**	**1.87 (1.27-2.75)**
		Frail	**2.35 (1.56-3.54)**	**1.28 (1.04-1.57)**	**3.30 (2.29-4.76)**
**CHS Criteria**^**1**^	Unadjusted	Prefrail	1.04 (0.59-1.81)	1.10 (0.84-1.43)	1.54 (0.95-2.51)
**N = 927**		Frail	**2.25 (1.40-3.61)**	**1.60 (1.27-2.02)**	**2.21 (1.41-3.46)**
	Adjusted^2^	Prefrail	0.88 (0.51-1.54)	1.06 (0.82-1.38)	1.49 (0.91-2.43)
		Frail	**1.74 (1.07-2.81)**	**1.45 (1.15-1.83)**	**2.17 (1.38-3.41)**
**CHESS Scale**	Unadjusted	Intermediate	**1.48 (1.04-2.09)**	**1.31 (1.10-1.56)**	1.33 (0.98-1.81)
**N = 1,066**		High	**2.13 (1.53-2.96)**	**1.32 (1.10-1.58)**	**1.84 (1.38-2.47)**
	Adjusted^2^	Intermediate	1.38 (0.98-1.94)	**1.27 (1.07-1.51)**	1.33 (0.98-1.82)
		High	**1.87 (1.35-2.59)**	**1.25 (1.05-1.50)**	**1.87 (1.39-2.50)**

*Robust (not frail or low risk for decline) used as reference category.

^1^For CHS analysis the number of events for death, hospitalization, and move to long-term care were 142, 375, and 172 respectively.

^2^Adjusted for age, sex, and co-morbidity (Charlson co-morbidity index score).

Frail and pre-frail individuals as defined by the Armstrong Index had no statistically significant increase in their risk for hospitalization (please see Table 3). Those meeting CHS criteria for frailty were at greater risk for hospitalization (risk ratio in adjusted model was 1.45, 95% CI 1.15-1.83), but there was no statistically significant increase in the risk ratio for the pre-frail group. Risk ratios for hospitalization were significantly increased for both pre-frail and frail subjects on the Full Frailty Index, but no gradient was observed and confidence intervals for the risk ratios for pre-frail (1.37, 95% CI 1.13-1.66) and frail (1.28, 95% CI 1.04-1.57) subjects overlapped. A similar increased risk for hospitalization was observed for those categorized as intermediate or high risk for decline on the CHESS scale in adjusted analyses.

Residents categorized as frail on all approaches considered and the high risk group for decline on the CHESS scale showed a statistically significant greater risk of transfer to long-term care (please see Table 3). The adjusted risk ratio was strongest for the Armstrong Frailty Index (4.14, 95% CI 1.87-9.14) followed by the Full Frailty Index (3.30, 95% CI 2.29-4.76). Only the Full Frailty Index showed a statistically significant increased risk of long term care admission for pre-frail individuals (1.87, 95% CI 1.27-2.75) in adjusted analyses.

To compare the abilities of the three frailty approaches and the CHESS scale to predict our outcomes of interest, we examined areas under the ROC curve (AUC). As potential confounders we considered sex, age, and co-morbidity index scores (please see Table 4). The AUCs obtained for the frailty measures and the CHESS scale ranged from 0.683-0.701 for mortality, 0.609-0.629 for hospitalization, and 0.602-0.667 for admission to long-term care within a year. For death, the addition of frailty (however operationalized) or CHESS-defined risk for decline significantly improved on the AUC obtained with sex, age, and co-morbidity (p-values less than 0.03 for pair-wise comparisons), though the magnitude of improvement seen was relatively small (0.031-0.049). None of the AUCs with a frailty measure or the CHESS scale differed significantly from each other. As for hospitalization, only the CHS frailty criteria significantly improved on the model based on age, sex, and co-morbidity (AUC 0.629, 95% CI 0.592-0.665 versus AUC 0.598, 95% CI 0.561-0.635, p = 0.003 for the comparison). The magnitude of improvement was relatively small (0.031), and none of the AUCs with the addition of frailty or the CHESS scale differed significantly from each other. For transfer to long-term care, the addition of any of the frailty measures or the CHESS-defined risk of decline improved on the model with sex, age and co-morbidity (p-values less than 0.03 for pair-wise comparisons). The magnitude of improvement seen ranged from 0.048 to 0.113. The AUC for the Full Frailty Index (0.667) differed significantly from the AUCs for the CHESS scale (0.602, p = 0.016) and CHS frailty criteria (0.610, p = 0.003). The difference between the AUCs of the Full Frailty Index and the Armstrong Index (0.638) was of borderline significance (p = 0.087).

**Table 4:** Area under the ROC curve (95% Confidence Intervals) for models of one-year outcomes, comparing frailty measures

**Model**		**Death**^**1**^	**Hospitalization**^**2**^	**Move to Long-Term care**^**1**^
1	Sex and age	0.638 (0.592-0.683)	0.531 (0.495-0.566)	0.552 (0.510-0.593)
2	Sex, age, and co-morbidity	0.652 (0.607-0.698)	0.592 (0.558-0.627)	0.554 (0.512-0.596)
3	Sex, age, co-morbidity, and **Armstrong Index**	0.683 (0.639-0.728)	0.609 (0.575-0.643)	0.638 (0.598-0.678)
4	Sex, age, co-morbidity, and **Full Frailty Index**	0.691 (0.648-0.733)	0.610 (0.576-0.644)	0.667 (0.625-0.707)^3^
5	Sex, age, co-morbidity, and **CHS Frailty Criteria**^**4**^	0.701 (0.655-0.747)	0.629 (0.592-0.665)	0.610 (0.564-0.656)
6	Sex, age, co-morbidity, and **CHESS Scale**	0.683 (0.640-0.725)	0.610 (0.576-0.645)	0.602 (0.558-0.646)

Note:

1. Models 3–6 differed from models 1 and 2 for outcomes of death and long-term care. All p-value less than 0.03.

2. For hospitalization outcome, models 3, 4, and 6 differed from model 1 (p <; .001 for all), and model 5 differed from both models 1 and 2 (p <; .001, p = .003), respectively).

3. For long-term care, model 4 differed from models 5 (p = .003), 6 (p = .016), and marginally with model 3 (p = .087).

4. All pair-wise comparisons involving model 5 are based on n = 927 residents; otherwise, n = 1066 residents. The relevant AUC (95%CIs) estimates for Models 1 and 2 for n = 927 residents are as follows:

Death: Model 1, 0.649 (0.600-0.698); Model 2, 0.670 (0.622-0.719).

Hospitalization: Model 1, 0.520 (0.481-0.558); Model 2, 0.598 (0.561-0.635).

Long-Term Care: Model 1, 0.552 (0.506-0.598); Model 2, 0.552 (0.506-0.598).

As a sensitivity analysis, we looked also at two composite outcomes (hospitalizations + deaths and long-term care transfers + deaths) to see if censoring affected our results. As they were essentially unchanged, we have not reported the specific estimates for these composite outcomes.

## Discussion

In this population of AL residents, both the presence of frailty defined by any of the three approaches examined and a higher CHESS score were associated with an increased risk of dying or being transferred to a long-term care facility. The magnitude of the ability of these measures to correctly classify subjects (relative to models including only age, sex and co-morbidity) was modest for mortality but better for long-term care placement. The AUCs obtained were in the 0.6 to 0.7 range. This generally indicates low accuracy in correctly differentiating risk [27], but our objective was not to develop a comprehensive prediction model for these outcomes in our AL population. The current study comparing these three frailty measures and the CHESS score builds directly on our prior work examining frailty in the AL setting [14].

The different approaches to detecting frailty were more similar than dissimilar with regard to predictive accuracy with a few exceptions. The Full Frailty Index performed significantly better in predicting a move to long-term care and was the only approach that showed higher mortality, hospitalization, and institutionalization risks among those categorized as pre-frail. Only the addition of CHS frailty criteria significantly improved on age, sex, and co-morbidity in predicting hospitalization. While our study did not directly address practicality, acceptability to practitioners, or clinical utility, a number of observations can be made. Both the Armstrong Index and CHS frailty criteria categorized relatively few AL residents as not frail and approximately half as frail. In comparison to the Full Frailty Index and the CHESS scale, they were less successful in creating a more circumscribed group of AL residents for potential interventions. This would be an important consideration when clinical resources are limited and have to be targeted. The Armstrong and Full Frailty Indices (and the CHESS scale) were constructed using data available on the interRAI-AL instrument, one of a suite of similar instruments being implemented across continuing care settings in Canada. While many items were included in the indices, their calculation could be automated. This could make the use of multi-item indices practical from a clinical standpoint. The CHS frailty criteria did not perform significantly better than the frailty indices (other than possibly in predicting hospitalization) and required the use of performance measures, which can be difficult to obtain on AL residents [14,28]. The latter issue raises major feasibility concerns with this approach in an AL population. The ability to derive the CHESS scale and two frailty indices from previously collected data highlights the practical utility of these measures while the inclusion of a wider set of potentially relevant domains in operationalizing frailty represents a conceptual strength of the two frailty indices [6].

As shown in Table 2, the best agreement among the measures was between the Full Frailty and Armstrong indices. Although moderate agreement between these two indices was observed [29], it is surprising that this was not greater given the degree of commonality in items (see Appendix A) and their similar operational approaches. Interestingly, Rockwood et al. found that random combinations of items making up frailty indices led to little overlap across quartiles [30]. The lower than expected level of agreement between these two indices may also reflect the inclusion of several Instrumental ADLs (e.g., meal preparation, housework, managing medications) in the Armstrong (but not the Full Frailty) index. As these areas are generally managed or provided for residents by the AL facility (resulting in most residents being assessed as impaired in these areas) they would be considered poor criteria for a frailty measure [16]. This may also explain the low proportion of residents categorized as not frail by the Armstrong index. Fair agreement was observed between the Full Frailty Index and the CHESS and between the Armstrong Frailty Index and the CHS. The remaining kappa statistics indicated only slight agreement between the measures. Armstrong et al. had previously reported a low correlation between the CHESS and their frailty index (r = 0.35) [10].

Whether the CHESS scale is a frailty measure cannot be answered by our study. Compared with the other measures examined, the CHESS scale generally performed similarly in predicting our outcomes of interest, although associations were weaker for long term care placement. This was most evident for comparisons between CHESS and the two frailty indices. In settings where interRAI instruments are widely implemented, the CHESS scale offers the advantage of being relatively simple and easy to assess across care settings.

The reasons underlying the relatively weaker associations between frailty and the outcome of hospitalization are not entirely clear. When added to the model with age and sex, the Charlson co-morbidity index score improved the AUC for hospitalization (see Table 4) and has been previously shown to predict hospitalization (and mortality) in older institutionalized residents [31]. The further addition of frailty and/or health instability may offer relatively less predictive gain for this outcome after the addition of a co-morbidity index. Another possibility is competing risk though we found no evidence of this in the analyses performed for this study. In earlier work we failed to observe a statistically significant increase in the risk of hospitalization among frail men that we felt was due to their high mortality rate during follow-up [14]. Other considerations would include the inherent difficulty of predicting hospitalizations especially for catastrophic (e.g., fall with a hip fracture) changes in health, the modifying effects of factors such as advance planning and the resources available within AL facilities [32], and the obscuring influence of variability in local hospitalization rates [33].

There are some limitations in our study that would raise concerns about the generalizability of our findings to the entire AL population. First, over four hundred eligible residents did not enroll in the study [14]. While the age and sex distribution of those not enrolled was comparable to our enrolled cohort, we do not have other information on them. Second, we restricted eligibility to residents of publicly-subsidized AL facilities in Alberta. Our findings may not apply to residents in private AL or AL-type facilities in other jurisdictions that may have different admission criteria, staffing, and institutional policies. Hospitalizations were determined using provincial data, and we may have missed the rare event that occurred outside Alberta. Further research examining these and other relevant outcomes (e.g., functional decline) over longer periods of time is also warranted. Nearly 40% of AL participants could not complete the CHS frailty assessment as originally intended. By using responses to observed items from the interRAI-AL assessment instrument we were able to reduce the proportion with missing data to 15%. As noted previously this raises questions about the feasibility of this particular approach in an AL population.

## Conclusions

Using a number of approaches, varying degrees of frailty can be detected within AL populations. Residents with more severe levels of frailty are more likely to die, be hospitalized, and require admission to long-term care. Whether the detection of frailty will turn out to be clinically useful will require further work, in particular intervention studies.

## Appendix A

The following items were included in the cumulative deficit index meant to replicate that created by Armstrong et al. [10], (Table 5). Three items which were not exactly replicable using the interRAI-AL were, ”severe malnutrition”, “problem chewing” and “head trauma”. Also “Alzheimer” and “Dementia other than Alzheimer’s” were combined into one category. “Hip fracture”, “other fractures”, and “osteoporosis” were combined into one category. Also, “Feeling of sadness” and “Sad, pained worried facial expressions” were combined into one category. This reduced the index from 50 items to 43 items.

The presence of each condition added “1” to the person’s Index score (unless otherwise indicated).

The following index was created using the criteria based on Searle et al. [16] using information from the interRAI-AL, (Table 6). The presence of each condition added “1” to the person’s index score (unless otherwise indicated).

The following details the frailty criteria from the Cardiovascular Health Study (CHS) [9], (Table 7).

**Table 5:** Armstrong frailty index (43 items)

**Name of frailty item by subject header**
**Mood**
Persistent anger
Unrealistic fears
Repetitive health complaints
Sad, pained, worried facial expressions
Withdrawal from activities of interest
Reduced social interactions
**Communication**
Moderate/severe vision problems
**Functional status**
Limited help (0.5), extensive help (1) with meal preparation
Limited help (0.5), extensive help (1) with ordinary housework
Limited help (0.5), extensive help (1) with managing finances
Limited help (0.5), extensive help (1) with managing meds
Limited help (0.5), extensive help (1) with stair climbing
Limited help (0.5), extensive help (1) with shopping
Limited help (0.5), extensive help (1) with bathing
Limited help (0.5), extensive help (1) with personal hygiene
Limited help (0.5), extensive help (1) with dressing upper body
Limited help (0.5), extensive help (1) with dressing lower body
Limited help (0.5), extensive help (1) with locomotion
Limited help (0.5), extensive help (1) with transferring
Minimal help (0.5), extensive help (1) with toilet use
Minimal help (0.5), extensive help (1) with eating
**Incontinence**
Some (0.5) or daily (1) bladder incontinence
**Disease diagnosis**
Hip fracture, other fractures, osteoporosis
Arthritis
Alzheimer’s disease/dementia
Hemiplegia
Multiple sclerosis
Parkinson's disease
Stroke or CVA
Hypertension
Coronary heart disease
Congestive heart failure
Chronic Obstructive Pulmonary Disease (COPD)/Emphysema/Asthma
Diabetes
Renal disease
Peripheral vascular disease
Cardiac arrhythmias
Thyroid disease
**Health conditions**
Balance - unsteady gait
Poor self-reported health
Unstable health
**Nutrition/Medications**
BMI 30–40 (0.5), BMI > 40 (1)
Weight loss 5% or more in last 30 days or 10% in last 180 days

**Table 6:** Full frailty index (83 items)

**Name of frailty item by subject header**
**Psychosocial well-being**
Not close to someone in facility
No strong supportive relationship with family
Infrequent participation in longstanding social activities
Infrequent visits from friends/family
Infrequent interaction with friends/family
**Mood**
Makes negative statements
Persistent anger
Unrealistic fears
Repetitive health complaints
Repetitive anxiety
Sad, pained, worried facial expressions
Crying, tearfulness
Withdrawal from activities of interest
Reduced social interactions
Lack of pleasure in life
**Cognition**
Minimally impaired (0.5) or moderate/severely impaired (1) decision-making skills
Short-term memory problems
Procedural memory problems
Situational memory problems
Easily distracted
Episodes of disorganized speech
Declined decision-making last 90 days
**Communication**
At least some difficulty to make self understood
At least some difficulty understanding
Moderate/severe hearing problems
Moderate/severe vision problems
**Functional status**
Limited help (0.5), extensive help (1) with phone use
Limited help (0.5), extensive help (1) with stair climbing
Limited help (0.5), extensive help (1) with shopping
Limited help (0.5), extensive help (1) with bathing
Limited help (0.5), extensive help (1) with personal hygiene
Limited help (0.5), extensive help (1) with dressing upper body
Limited help (0.5), extensive help (1) with dressing lower body
Limited help (0.5), extensive help (1) with walking
Limited help (0.5), extensive help (1) with locomotion
Limited help (0.5), extensive help (1) with transferring
Minimal help (0.5), extensive help (1) with toilet use
Minimal help (0.5), extensive help (1) with bed mobility
Minimal help (0.5), extensive help (1) with eating
Less than 1 hour of physical activity in last 3 days
Did not go out within a 3 day period
Declined in ADL over last 90 days
**Incontinence**
Some (0.5), daily (1) bladder incontinence
Some (0.5), daily (1) bowel incontinence
**Disease diagnosis**
Hip fracture, other fractures, osteoporosis
Arthritis
Alzheimer’s disease/dementia
Hemiplegia
Multiple sclerosis
Paraplegia/quadriplegia
Parkinson's disease
Stroke or CVA
Hypertension
Coronary heart disease
Congestive heart failure
COPD/Emphysema/Asthma
Cancer
Diabetes
Renal disease
Peripheral vascular disease
Cardiac arrhythmias
Thyroid disease
**Health conditions**
At least 1 fall in last 30 days
Balance - turning around
Balance - dizziness
Balance - unsteady gait
Chest pain
Abnormal thought process
Delusions
Hallucinations
Aphasia
Vomiting
Non-restful sleep/insomnia
Too much sleep
Peripheral edema
Shortness of breath
Fatigue - cannot complete day-to-day activities
Pain present
Poor self-reported health
**Nutrition/Medications**
BMI 30–40 (0.5), BMI > 40 (1)
Weight loss 5% or more in last 30 days or 10% in last 180 days
Ten or more medications
Allergy to any drug

**Table 7:** Cardiovascular Health Study Frailty Criteria^1^

**Criterion**	**Measure**	**Frailty criteria**
*Slow gait*	Determined by taking the better of two timed 3-meter walks.	≥7 seconds^2^, men ≤ 173 cm ≥ 7 seconds, women ≤ 159 cm ≥ 6 seconds, men > 173 cm ≥ 6 seconds, women > 159 cm
*Muscle weakness*	Average of three grip strength readings using a handheld dynamometer.^3^	BMI-specific thresholds: ≤ 29-32 kg, men ≤ 17-21 kg, women
*Low physical activity*	Reported minutes over two weeks per activity type - from the inter*RAI-AL “*Exercise or Leisure Activities” ^4^	Activities were mapped to Minnesota Leisure Time Activity Questionnaire [34]. Kcals per week calculated based on the intensity codes: < 383 Kcals/week, men < 270 Kcals/week, women
*Unintentional weight loss*	Answer to question: “In the past year have you lost more than 10 pounds unintentionally” ^5^	Response of “Yes”
*Exhaustion*	Answers to 3 questions: “In the past month, on average, have you been: 1) Feeling unusually tired during the day?; 2) Feeling unusually weak?; and/or, 3) Feeling an unusually low energy level?”^6^	Response of Yes to any of the 3 questions

*CHS* Cardiovascular Health Study, *AL* assisted living, *cm* centimeters, *BMI* body mass index, *kg* kilograms, *kcals* kilocalories.^1^As detailed in Fried et al. 2001 [9].^2^Sex and height-specific thresholds.^3^JAMAR®, Sammons Preston Rolyan, Bolingbrook, IL.^4^Include: aquasize/swimming; bowling; dancing; exercise bike/treadmill; exercise program; floor curling/lawn bowling; gardening; household chores; shuffleboard/pool; Tai chi/yoga; walking/wheeling indoors & outdoors.^5^CHS also allowed actual unintentional 5% weight loss over 1-year (not assessed in ACCES).^6^CHS used 2 items from the CES-D Scale [35]: “I feel that everything I do is an effort” and “I cannot get going” (those reporting feeling this way at least 3–4 days/previous week fulfilled the criterion).

## Appendix B

### CHESS Scale

CHESS stands for Changes in Health, End-stage disease and Symptoms and Signs of medical problems [11]. It provides a measure of instability in health (which may be a consequence of frailty) and is believed to be a marker of imminent decline in health. The score is based on the following:

Symptoms: A summary count is first derived from the following symptoms (coded as 0 = no symptoms present; 1 = 1 symptom present; 2 = 2+ symptoms present).

 • vomiting

 • dehydration (insufficient fluid)*

 • decline in food/fluid intake*

 • weight loss

 • shortness of breath

 • edema

To this summary count variable, 1 point is added for “worsening of decision making over previous 90 days”, 1 point for “decline in activities of daily living over previous 90 days”, and 1 point for “end-stage disease”.

The range of values for the CHESS is 0 to 5, where 0 represents stability, and 5 represents highly unstable health.

*Note that two items from the original CHESS, were unavailable on the interRAI-AL form, and were not included in the calculation of symptoms:

 • dehydration

 • decline in food/fluid intake

## Competing interests

The authors declare that they have no competing interests.

## Authors’ contributions

DH, EF and CM were responsible for the conception and design of the frailty study and for the initial interpretation of the data. DH drafted the initial manuscript and EF was responsible for the main analysis. LS and CM were responsible for the conception and design of the parent ACCES study, directed the acquisition of data and made substantial contributions to the analysis and interpretation of the data. SP, HS and DR provided significant input regarding the original frailty study design and clinical interpretation of the data. All authors were involved in revising the manuscript critically for important intellectual content and have given final approval of the version to be published.

## Pre-publication history

The pre-publication history for this paper can be accessed here:

http://www.biomedcentral.com/1471-2318/12/56/prepub
